# Linking mental health and digital addiction in general population: an overview of thematic evolution and trends from a science mapping perspective

**DOI:** 10.3389/fpsyt.2026.1806866

**Published:** 2026-05-08

**Authors:** Turgut Karaköse, Mehmet Ozdogru, Bünyamin Han, Tijen Tülübaş, Abdurrahman Kardas, Murat Demirkol, Hakan Polat

**Affiliations:** 1 Faculty of Education, Kütahya Dumlupınar University, Kütahya, Türkiye; 2 Provincial Directorate of National Education, Batman, Türkiye; 3 Faculty of Education, Firat University, Elazığ, Türkiye

**Keywords:** addictive disorders, digital addiction, internet addiction, mental disorders, mental health, social media addiction

## Abstract

**Introduction:**

Digital addiction, the problematic or addictive use of digital devices and platforms such as the internet, smartphone, or social media, harms people, including their mental health. Rapid developments in digital technologies have increased research interest in this relationship in diverse fields of study, accumulating a sound knowledge base.

**Methods:**

This study investigates this rapidly growing, multidisciplinary research field through bibliometric science mapping analysis and aims to offer an integrative framework for understanding how knowledge on digital addiction-mental health was built across these diverse fields. The metadata of 863 articles retrieved from Web of Science and Scopus were submitted to period-based, comparative analyses using SciMAT software.

**Results:**

Strategic themes, thematic network structures, and thematic evolution map showed that research investigating the digital addiction-mental health relationship focused on addictive behaviors (mostly with reference to the internet, social media, and smartphone addiction) and primarily investigated their relation to anxiety disorder, sleep quality, and depression.

**Discussion:**

By providing a systematic mapping of key concepts, their interconnections, and their evolution over time, the study identified emerging research fronts, underexplored populations, and neglected mental health outcomes and suggested significant implications for future research trajectories in the field.

## 1 Introduction

Today, digital technologies have become an indispensable part of daily life (1), making life more convenient (2, 3). Yet, research has also highlighted the negative effects associated with their excessive and problematic use (4–6), which can be defined as “digital addiction” in broader terms.

Used as an umbrella term referring to the excessive, uncontrolled use of digital technologies such as the internet, video games, online platforms, mobile devices, and social networks (7–9), digital addiction (digital addiction) is currently considered a public health problem worldwide (10). This disruptive use of digital technologies produces behavioral symptoms like the ones observed in any type of addiction (11). It can cause serious psychological problems (12). From this perspective, digital addiction is a form of behavioral addiction resulting from a maladaptive coping with anxiety, emotional distress, or stress.

Despite some counter-arguments that the concept of ‘addiction’ can present an overly pathologizing framework without first identifying the underlying motives and contexts of their problematic use (13), there is a growing consensus in the literature that digital addiction involves the core components identified for behavioral addictions: salience, mood modification, tolerance, withdrawal, conflict, and relapse (14), indicating that digital addiction is not merely a phenomenon of “intense interest” but rather represents a dysfunctional, problematic, and addictive relationship between the individual and the relevant digital technology (13).

Digital addiction involves several subtypes of addiction, such as generalized internet addiction, internet gaming disorder, social media addiction, and smartphone addiction (8). Each of these subtypes is associated with unique cognitive and emotional mechanisms. For example, social media addiction exhibits a usage pattern driven by “the reinforcement of social feedback loops and fear of missing out (15, p. 288). In contrast, smartphone addiction is associated with “the constant accessibility and instant gratification of app-based interactions” (13, p. 141). This underscores that digital addiction should not be considered a singular disorder, but a multidimensional concept that varies across different digital platforms and interactions (16).

Excessive exposure to digital technologies or their problematic use has raised serious concerns about the potential negative impacts on individuals’ physical and mental health, particularly through the development of digital addiction (17, 18). Studies have shown that problematic use of digital technologies or addictive behaviors regarding their use is highly correlated with various psychological problems such as depression, anxiety, and sleep disorders (19–30). These findings indicate that digital addiction is not only a problem on its own but is a significant risk factor for mental health.

The World Health Organization (31) defines mental health (mental health) as “a state of well-being that enables an individual to cope with the stresses of life, realize their own abilities, work productively, and contribute to society.” mental health encompasses an individual’s capacity to effectively cope with the normal stressors they encounter in daily life and to interact meaningfully with society (32). It has been increasingly recognized that, by weakening an individual’s psychological resilience and social functioning, digital addictive behaviors can be linked to mental health problems such as suicidal ideation, depression, stress, and anxiety symptoms (10, 33–37).

Following the rapid increase in digital technologies and their integration into the daily lives of people, concerns over their problematic use and addictive potential have instigated growing research interest into their potential threats to people’s mental health and psychological well-being, accumulating a significant volume of research from various research fields across psychology, psychiatry, public health, education, and information sciences. The current bibliometric science-mapping study was conducted to provide a comprehensive overview of this rapidly growing, multidisciplinary research field by identifying its thematic and intellectual evolution and offering an integrative framework for understanding how knowledge on digital addiction-mental health research was built across these diverse fields. By providing a systematic mapping of key concepts, their interconnections, and their evolution over time, the study identifies emerging research fronts, underexplored populations, and neglected mental health outcomes, thereby suggesting significant implications for future research trajectories in the field (38, 39).

Bibliometric science-mapping is widely recognized as a valuable approach for advancing understanding within a research field. By systematically analyzing patterns in the scholarly literature, this method helps identify both well-established and relatively underexplored areas of inquiry while clarifying the conceptual boundaries of existing knowledge. Ultimately, by synthesizing diverse strands of research and highlighting the emergence of different intellectual perspectives, bibliometric science-mapping can offer a robust foundation for guiding future research agendas and informing policy development (29, 40).

In addition to these contributions, the present study also offers a novel perspective into science-mapping by adopting the capabilities of SciMAT, which provides several methodological advantages over widely used bibliometric visualization tools. Unlike other tools, which usually provide cross-sectional snapshots of the intellectual structure of a research field, SciMAT is specifically designed to support longitudinal science-mapping analyses, enabling researchers to trace the development, transformation, and interconnection of research themes across successive time periods. These analyses further illustrate how knowledge grows, diffuses, and evolves across different periods through the examination of publication patterns over time. SciMAT is also capable of integrating performance indicators with strategic diagrams and thematic evolution maps, which helps identify several types of themes that might have guided the development of the field or might have remained weakly attended despite their potential for the field’s growth (41). By using SciMAT, the present study not only describes the existing structure of the digital addiction-mental health research domain but also helps interpret how research agendas evolved, consolidated, and shifted over time. Thus, the study facilitates a more systematic and theory-informed understanding of knowledge development, enabling the identification of emerging research fronts, conceptual gaps, and interdisciplinary linkages that may remain less visible in conventional bibliometric analyses.

## 2 Methods

This study was designed using bibliometric research methods to reveal the intellectual evolution of research addressing the digital addiction-mental health relationship. Using SciMAT software, different types of thematic strands were identified to demonstrate the field’s conceptual growth, and reflections were made on the thematic and intellectual evolution of the field across three periods of analysis, comprising 1981-2025.

### 2.1 Data collection and extraction

Data for this study were gathered through a simultaneous search on Web of Science (WoS) and Scopus, two databases indexing research from a wide variety of research fields, on September 13, 2025, using the following search string:


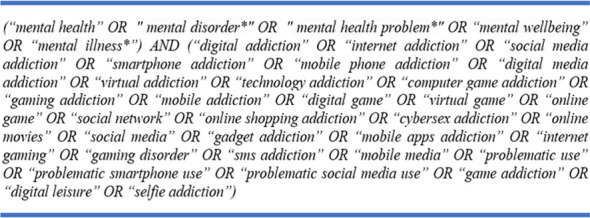
.

**Figure 1:**
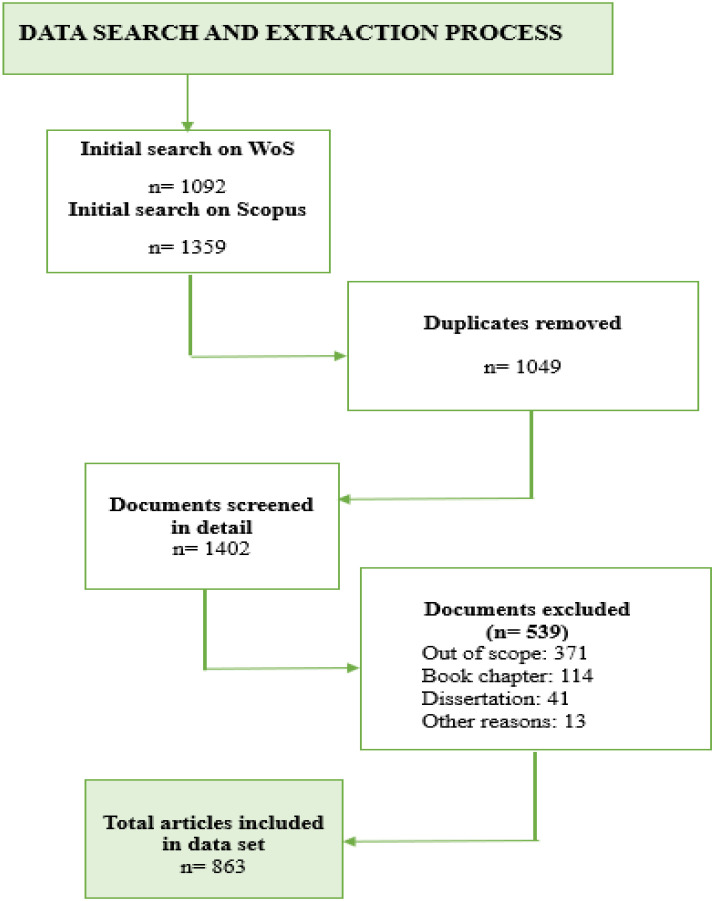
The PRISMA diagram.

The initial search returned 1092 documents from WoS and 1359 from Scopus. Following the Preferred Reporting Items for Systematic Reviews and Meta-Analyses (PRISMA) procedures, the data search and extraction process is depicted in the PRISMA diagram in **Figure 1**.

As shown in **Figure 1**, raw data returned by the initial search (n=2451) were first examined to identify the duplicates, and 1049 documents were removed as duplicates. The remaining 1402 documents underwent detailed screening to identify documents that were outside the study scope or did not provide eligible data for the analysis (i.e., books, book chapters, dissertations). Some documents were also removed because they were not in English, since all keywords included in the analysis must be in the same language for rigorous analysis. At the end of this extraction process, 539 documents were removed from the list, and 863 documents were included in the final dataset.

### 2.2 Data analysis and interpretation

Data analysis was conducted using SciMAT software. Because science-mapping analyses rely heavily on keyword pre-processing, we first carried out a keyword standardization process to ensure conceptual consistency in the analysis through a combination of automated cleaning functions within SciMAT and manual screening to ensure semantic accuracy. More specifically, we reconciled semantically similar but terminologically different keywords by merging synonymous terms (e.g., “smartphone addiction” and “mobile phone addiction”) under a unified label based on their conceptual equivalence. In addition, we standardized variations due to pluralization, spelling differences, and abbreviations (e.g., “behavior” vs. “behaviour”, “ICT” vs. “information and communication technologies”).

**Figure 2:**
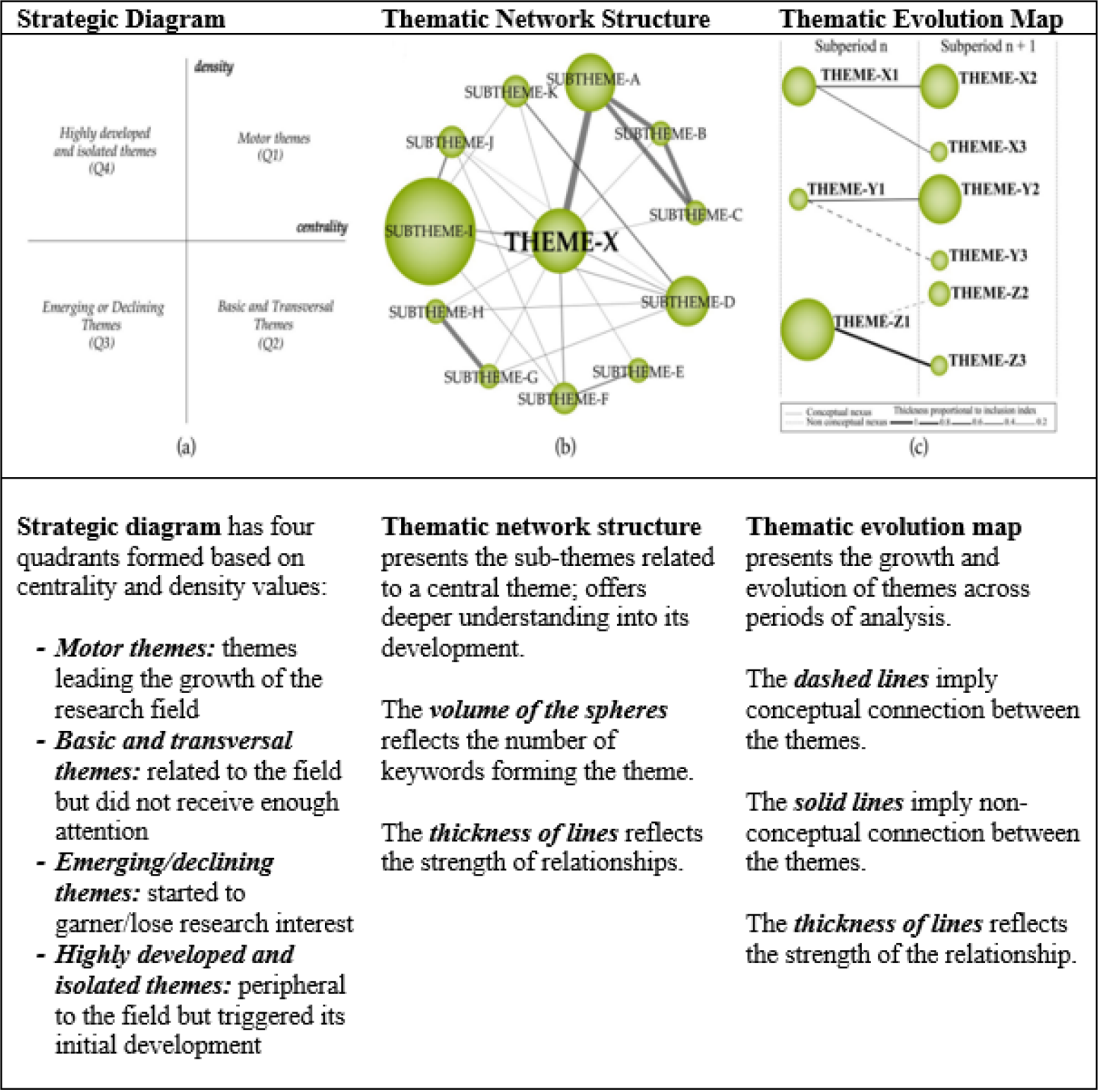
Guidelines for data analysis and interpretation (adapted from 42, and 43).

SciMAT analysis produces three maps that display the central themes of the field’s intellectual development across three periods of analysis: strategic diagram, thematic network structure, and thematic evolution map. Results of the science mapping analysis are interpreted using these maps, and guidelines for data interpretation are given in **Figure 2**.

The strategic diagram and thematic network structures are produced for each period of analysis. The strategic diagram presents four types of themes observed during the relevant period and explains their significance and contributions to the field’s growth. The thematic network structure maps thematic clusters, with the main theme at the center and relevant sub-themes around it, connected by lines. The thematic evolution map, on the other hand, comprises all three periods and demonstrates how themes evolved across them, thereby delineating a more comprehensive picture of the field’s intellectual evolution.

SciMAT not only allows for identifying several types of themes contributing to the development of a research field but also enables comparative analysis to determine how these themes were transformed over consecutive periods. Therefore, three periods of analysis were defined based on the accumulated number of publications in the dataset: Period 1 (1981-2019), Period 2 (2020-2022), and Period 3 (2023-2025).

## 3 Results and discussion

### 3.1 Publication trends

A total of 863 articles retrieved from the WoS and Scopus databases were analyzed using SciMAT. The findings from the thematic analysis are presented in strategic diagrams and thematic network clusters for each period of analysis. Findings from the thematic evolution analysis are presented in an overlapping graph delineating the evolution of themes across periods. Performance analyses involving h-index, citation count, centrality, and density values for the themes of each period are calculated and presented with strategic diagrams.

**Figure 3:**
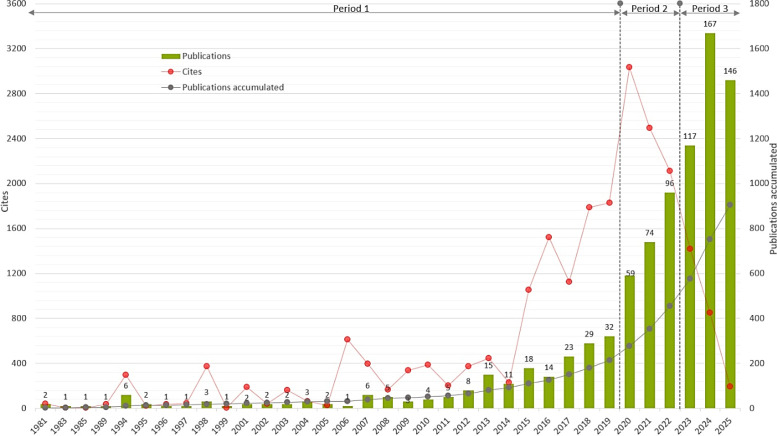
Publication trends (1981-2025).

Publication trends in the digital addiction-mental health research field across three periods of its development are presented in **Figure 3**.

As shown in **Figure 3**, the number of publications was limited until the mid-2000s but increased after 2010, following the emergence of various digital addiction types (e.g., smartphone, social media, or gaming addiction). Parallel to the steady rise in the number of publications, the number of citations gradually increased over the year, especially after 2015. These results indicate that Period 1 comprised the growth phase of the research field, marked by a sharp increase in research volume as early as Period 2 (2020–2022). This growth coincides with increased research interest in behavioral changes such as increased screen time, mandatory online education, and social isolation, particularly with reference to mental health, during/following the COVID-19 pandemic. Both the annual number of publications and citations reached historically high levels during Period 2. This increasing trend continued during Period 3 (2023–2025). Given the highest publication volume, particularly in 2023 and 2024, and the cumulative publication curve rising rapidly, it is evident that research interest in the digital addiction-mental health relationship has acquired a permanent, systematic, and multidisciplinary structure during Period 3, marking the maturation phase of this research field. Overall, the graph reveals that the mental health and digital addiction research field has become stronger, especially during the post-pandemic period, and the significant increase in publication and citation trends clearly reflects the growing interest in understanding the effects of digitalization on mental health.

An analysis of the geographical distribution of the literature was also conducted, considering that digital technology use and addiction phenomena can be deeply influenced by country context, and thus, the analysis of the most productive countries or regions can provide a more nuanced global perspective. The map for the geographical distribution of the digital addiction-mental health research is given in **Figure 3**.

**Figure 4:**
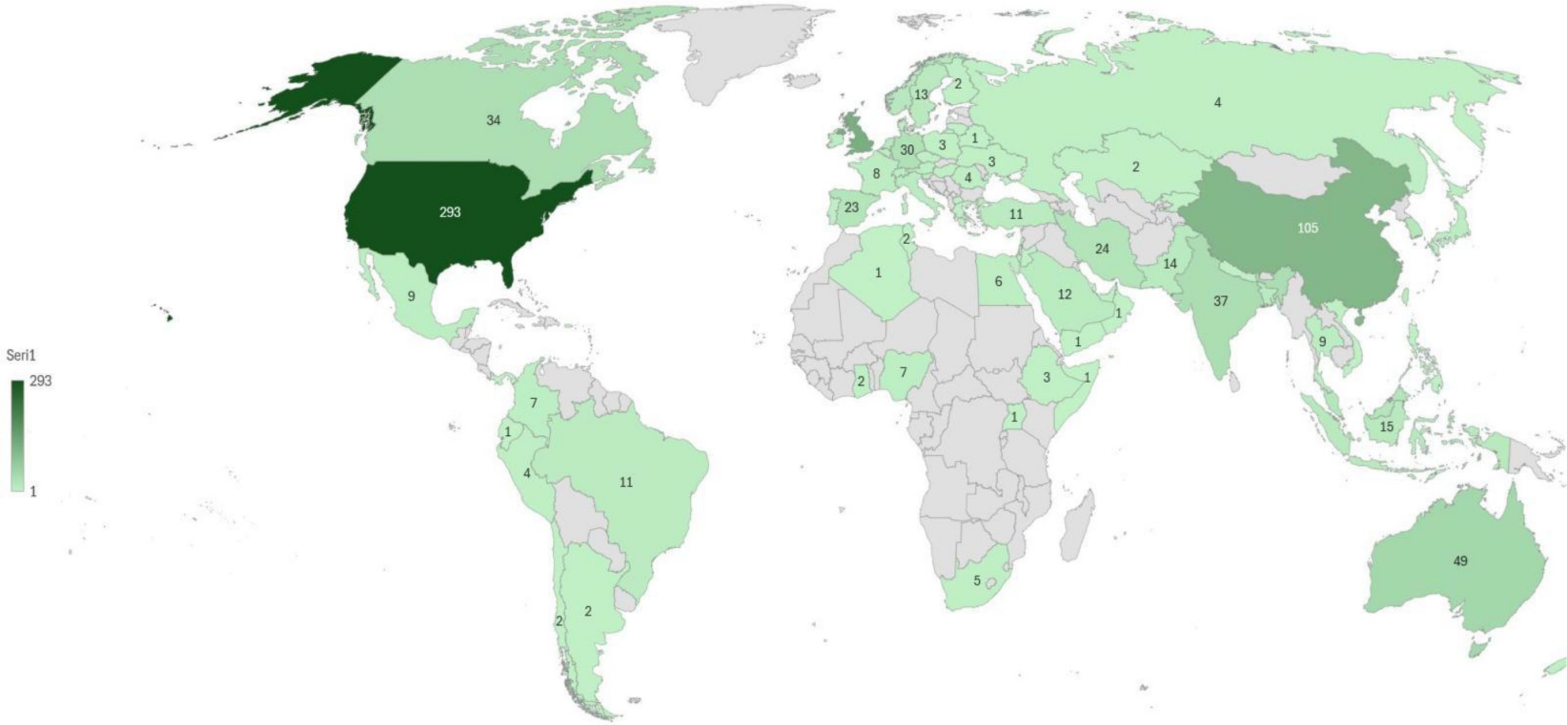
The geographical distribution of the digital addiction-mental health research.

As can be seen in **Figure 4**, the geographic distribution of research on digital addiction-mental health research reveals a clear concentration of scholarly activity in North America, East Asia, and parts of Europe and Australia. The United States leads the field, followed by China, Australia, and India, indicating that research was primarily driven by high-income countries while moderate contributions were made by several European nations. However, Africa, South America, and much of the Middle East exhibit comparatively limited output. This uneven distribution suggests that the existing literature may reflect region-specific perspectives and highlights opportunities for greater research engagement in underrepresented regions.

### 3.2 Thematic structure analysis

#### 3.2.1 Period 1 (1981-2019)

**Figure 5:**
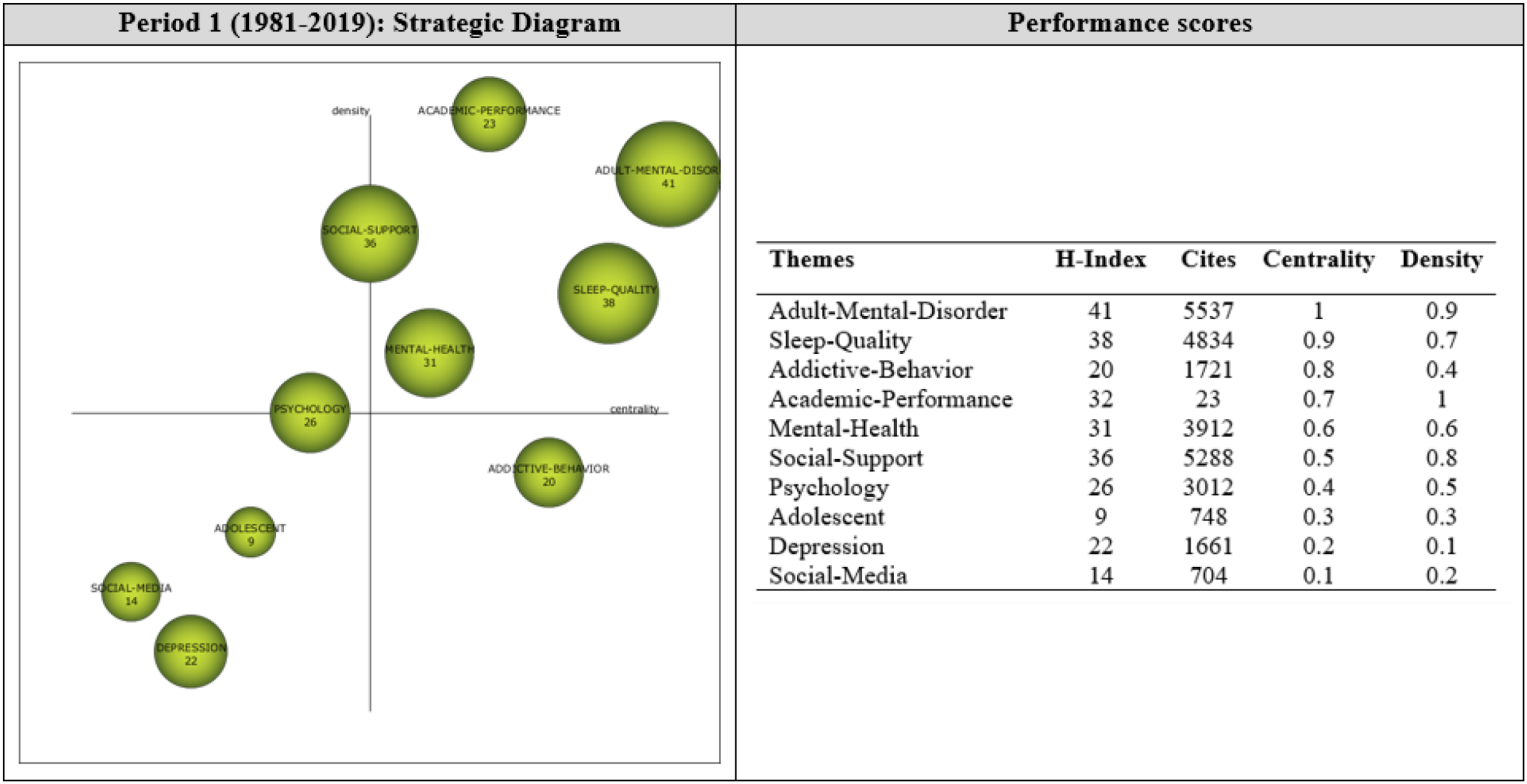
Strategic themes of Period 1 (1981-2019).

**Figure 6:**
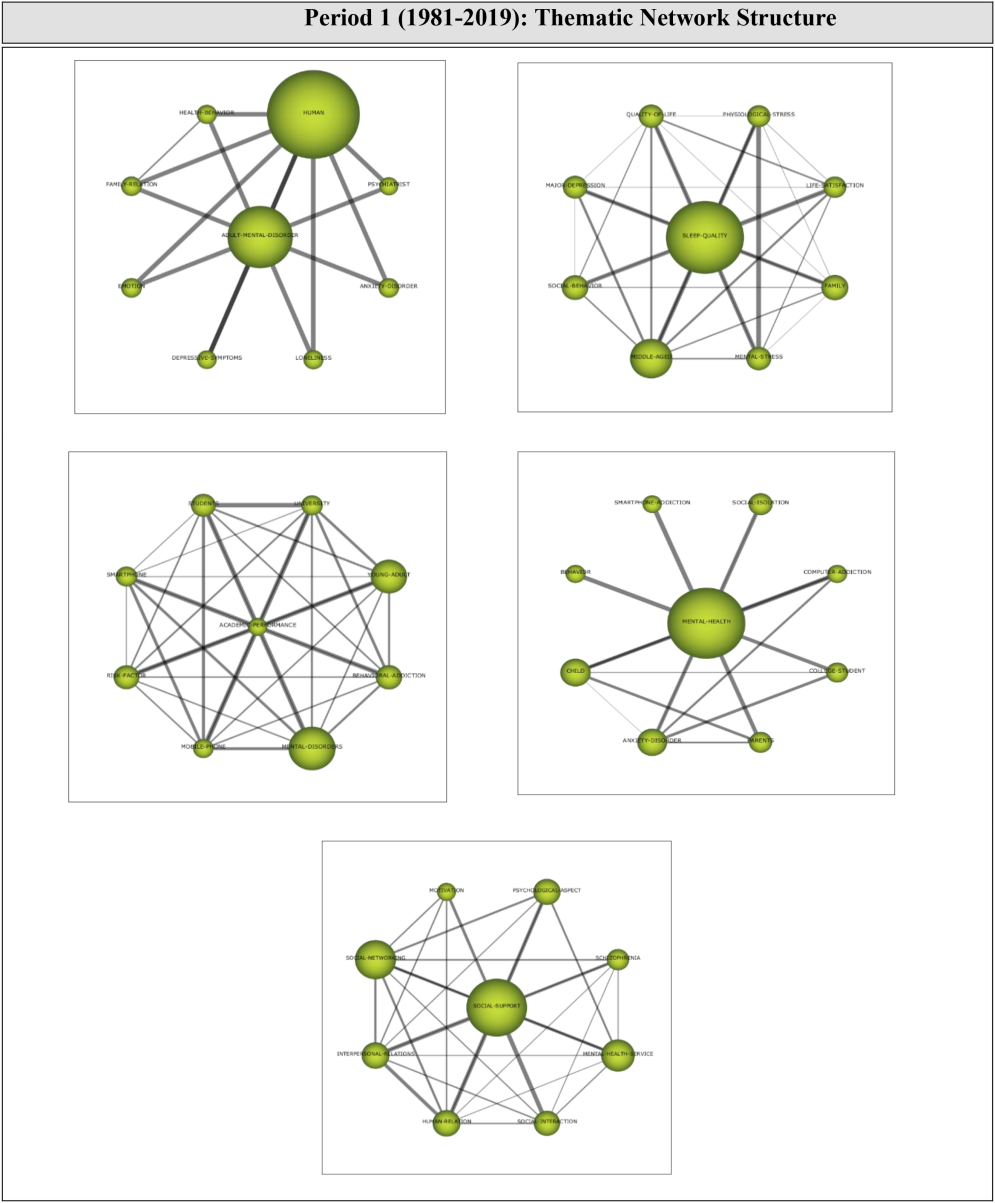
Thematic cluster networks for Period 1 (1981-2019) motor themes.

Analysis of 204 articles published during Period 1 revealed 10 themes that guided research during this period. As shown in **Figure 5**, the most significant themes (i.e., the motor themes) were “Adult-Mental-Disorder”, “Sleep-Quality”, “Academic-Performance”, “Mental-Health”, and “Social-Support”. Thematic cluster analyses were also conducted to reveal a network of sub-themes that generated these motor themes (**Figure 6**) and to reflect more deeply on their growth and influence.

Analysis of documents from Period 1 (1981-2019) revealed five motor themes, ADULT-MENTAL-DISORDER, SLEEP-QUALITY, ACADEMIC-PERFORMANCE, MENTAL-HEALTH, and SOCIAL-SUPPORT, which had high centrality and intensity values, and thus contributed to the growth of the mental health-digital addiction research field during this period. The PSYCHOLOGY theme was found to be a highly developed and isolated theme, indicating that the theme did not have the appropriate background or significance for the development of the field during this period. The ADOLESCENT, SOCIAL-MEDIA, and DEPRESSION themes appeared to be emerging/declining themes, which can be considered as emerging themes during the period, as it marks the beginning of this research field. The ADDICTIVE-BEHAVIOR theme had high centrality and low intensity values, and thus was located in the basic and transversal themes quartile, which involves themes that are important for the development of the field but did not receive sufficient attention during the period. The theme with the highest H-index during this period was the ADULT-MENTAL-DISORDER theme.

Thematic network structures (networks of clusters) were examined to determine the sub-themes underlying the growth of the motor themes during Period 1 (1981-2019). The thematic network of each motor theme is shown in **Figure 6**.

The motor theme ADULT-MENTAL-DISORDER (1, 0.9) was found to be strongly related to the sub-themes of *Human, Psychiatrist, Anxiety-Disorder, Loneliness, Depressive-Symptoms, Emotion, Family-Relation*, and *Health-Behavior*. Some sub-themes were also found to be related to each other. These results indicate that the research field focused on topics such as mental disorders in adults, loneliness, and family relationships during this period.

The motor theme SLEEP-QUALITY (0.9, 0.7) was found to be strongly related to the sub-themes of *Physiological Stress, Life Satisfaction, Family, Mental Stress, Middle-Aged, Social Behavior, Major Depression*, and *Quality of Life*. The themes within the cluster network were found to be interrelated. These results indicate an increased research focus on some factors related to sleep quality.

The motor theme ACADEMIC-PERFORMANCE (0.7, 1) was found to be related to the sub-themes of *University, Young-Adult, Behavioral-Addiction, Mental-Disorders, Mobile-Phone, Risk-Factor, Smartphone*, and *Students.* The sub-themes were also related to each other, indicating that this line of research focused on the effect of mental disorders on academic performance, with a particular interest in smartphone use.

The motor theme MENTAL-HEALTH (0.6, 0.6) was found to be related to the sub-themes of *Social-Isolation, Computer-Addiction, College-Student, Parents, Anxiety-Disorder, Child, Behavior*, and *Smartphone-Addiction*. It was also observed that some sub-themes were interrelated. These results show that research on students’ mental health has focused on smartphone and computer addiction.

The motor theme SOCIAL-SUPPORT (0.5, 0.8) was found to be related to the sub-themes of *Psychological Aspect, Schizophrenia, Mental Health Service, Social Interaction, Human Relations, Interpersonal Relations, Social Networking*, and *Motivation.* All sub-themes were found to be interrelated, indicating that focus on mental health shifted towards social support issues, including human relations, interaction, and motivation.

#### 3.2.2 Period 2 (2020-2022)

**Figure 7:**
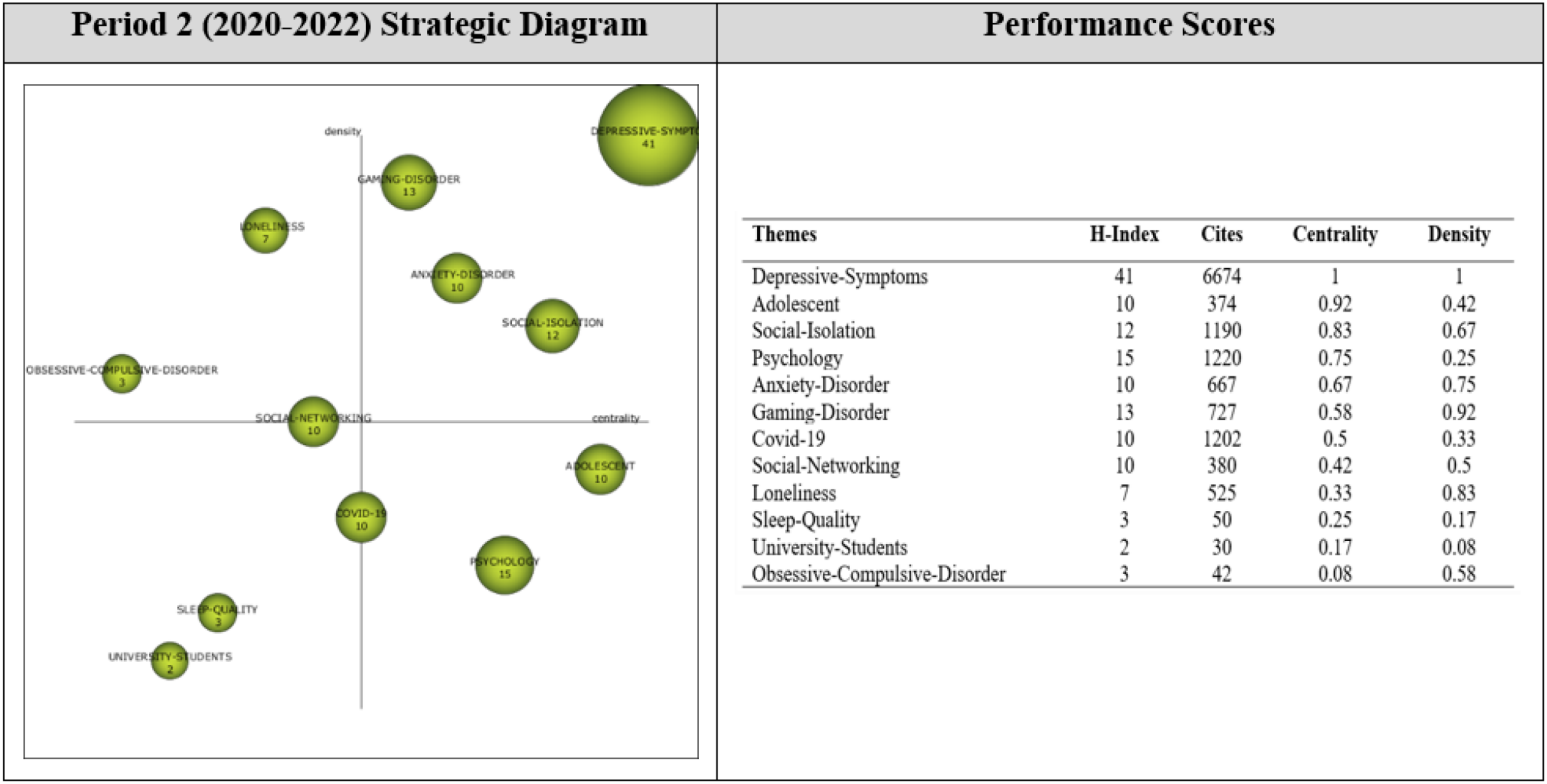
Strategic themes of Period 2 (2020-2022).

Analysis of 229 articles published during Period 2 revealed 12 themes that guided research during this period. As shown in **Figure 7**, the motor themes driving the growth of this research field during the second period were found to be DEPRESSIVE-SYMPTOMS, SOCIAL-ISOLATION, ANXIETY-DISORDER, and GAMING-DISORDER. The themes of LONELINESS, OBSESSIVE-COMPULSIVE-DISORDER, and SOCIAL-NETWORKING were highly developed and isolated themes, remaining peripheral to the development of the field, as they did not have a suitable background or significance for the research field. The SLEEP-QUALITY and UNIVERSITY-STUDENTS themes were in the emerging/declining themes quartile for either losing or garnering research interest during the period. The ADOLESCENT, COVID-19, and PSYCHOLOGY themes had high centrality and low intensity values, and were placed in the basic and transversal themes quartile, indicating that these themes did not receive sufficient research interest despite their significant potential to support the growth of the field. The theme with the highest H-index during this period was DEPRESSIVE-SYMPTOMS.

**Figure 8:**
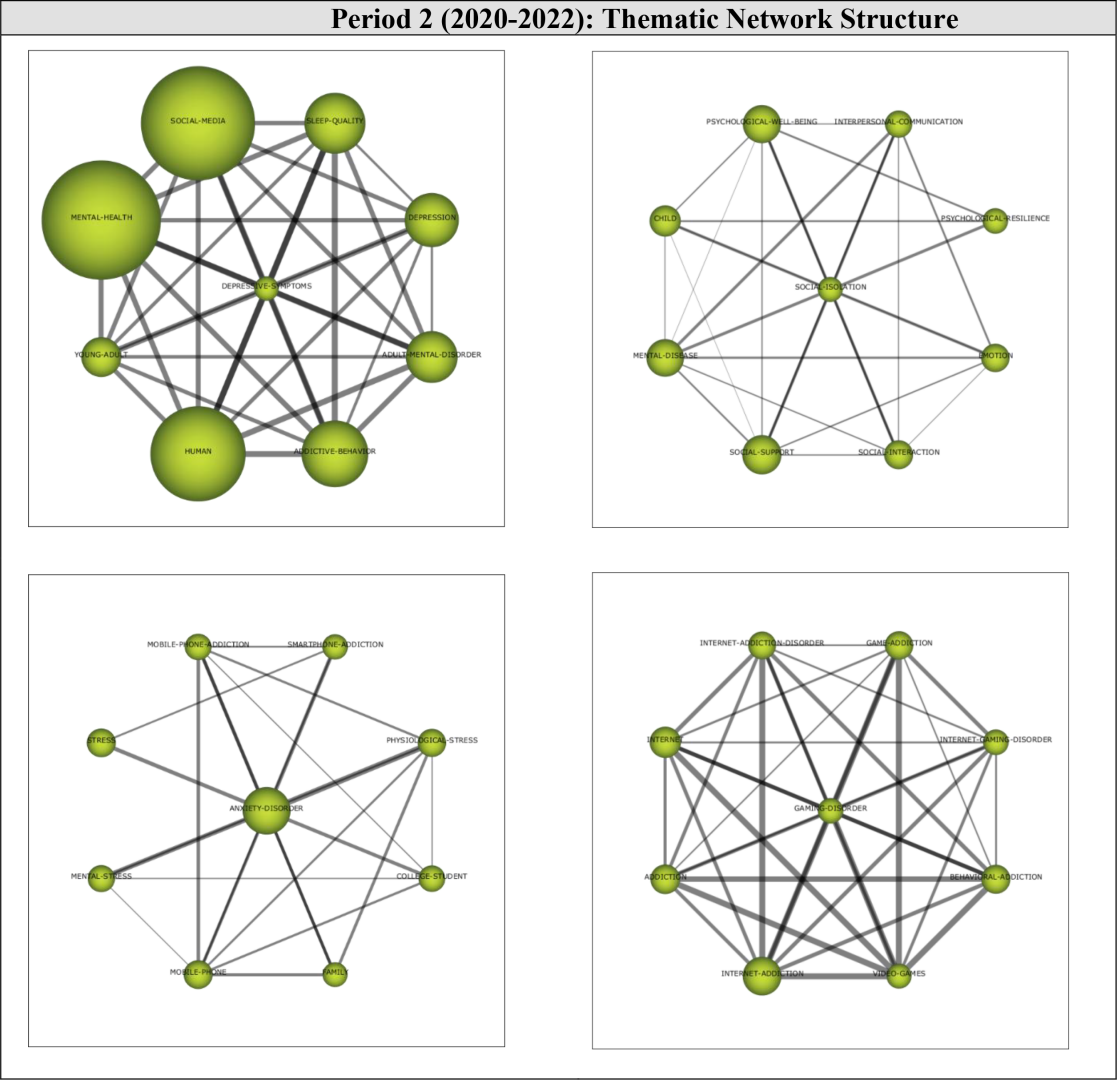
Thematic cluster networks for Period 2 (2020-2022) motor themes.

Thematic network structures were examined to determine the relevant sub-themes that supported the growth of the motor themes during the second period (2020-2022). The thematic network of each motor theme is shown in **Figure 8**.

The motor theme DEPRESSIVE-SYMPTOMS (1, 1) was found to have strong relationships with the sub-themes of *Sleep-Quality, Depression, Adult-Mental-Disorder, Addictive-Behavior, Human, Young-Adult, Mental-Health*, and *Social-Media.* All sub-themes were found to be interrelated. These results indicate that studies on mental health-digital addiction focused on the effects of social media use, such as mental health issues, depression, and sleep quality.

The motor theme SOCIAL-ISOLATION (0.83, 0.67) was found to have strong relationships with the sub-themes of *Interpersonal-Communication, Psychological-Resilience, Emotion, Social-Interaction, Social-Support, Mental-Disease, Child*, and *Psychological-Well-Being.* All sub-themes were found to be interrelated. These results indicate that this line of research focused on social support, social interaction, and interpersonal communication during this period.

The motor theme ANXIETY-DISORDER (0.67, 0.75) was found to be strongly related to the sub-themes of *Smartphone-Addiction, Physiological-Stress, College-Student, Family, Mobile-Phone, Mental-Stress, Stress*, and *Mobile-Phone-Addiction*, all of which were interrelated. These results suggest that research focused on the relationship between smartphones, physiological stress, and mental stress in anxiety disorders.

The motor theme GAMING-DISORDER (0.58, 0.92) was found to be related to the sub-themes of *Game-Addiction, Internet-Gaming-Disorder, Behavioral-Addiction, Video-Games, Internet-Addiction, Addiction, Internet*, and *Internet-Addiction-Disorder.* All sub-themes were found to be interrelated. These results indicate that the internet and gaming addiction garnered the interest of researchers investigating the digital addiction-mental health relationship during this period.

#### 3.2.3 Period 3 (2023-2025)

**Figure 9:**
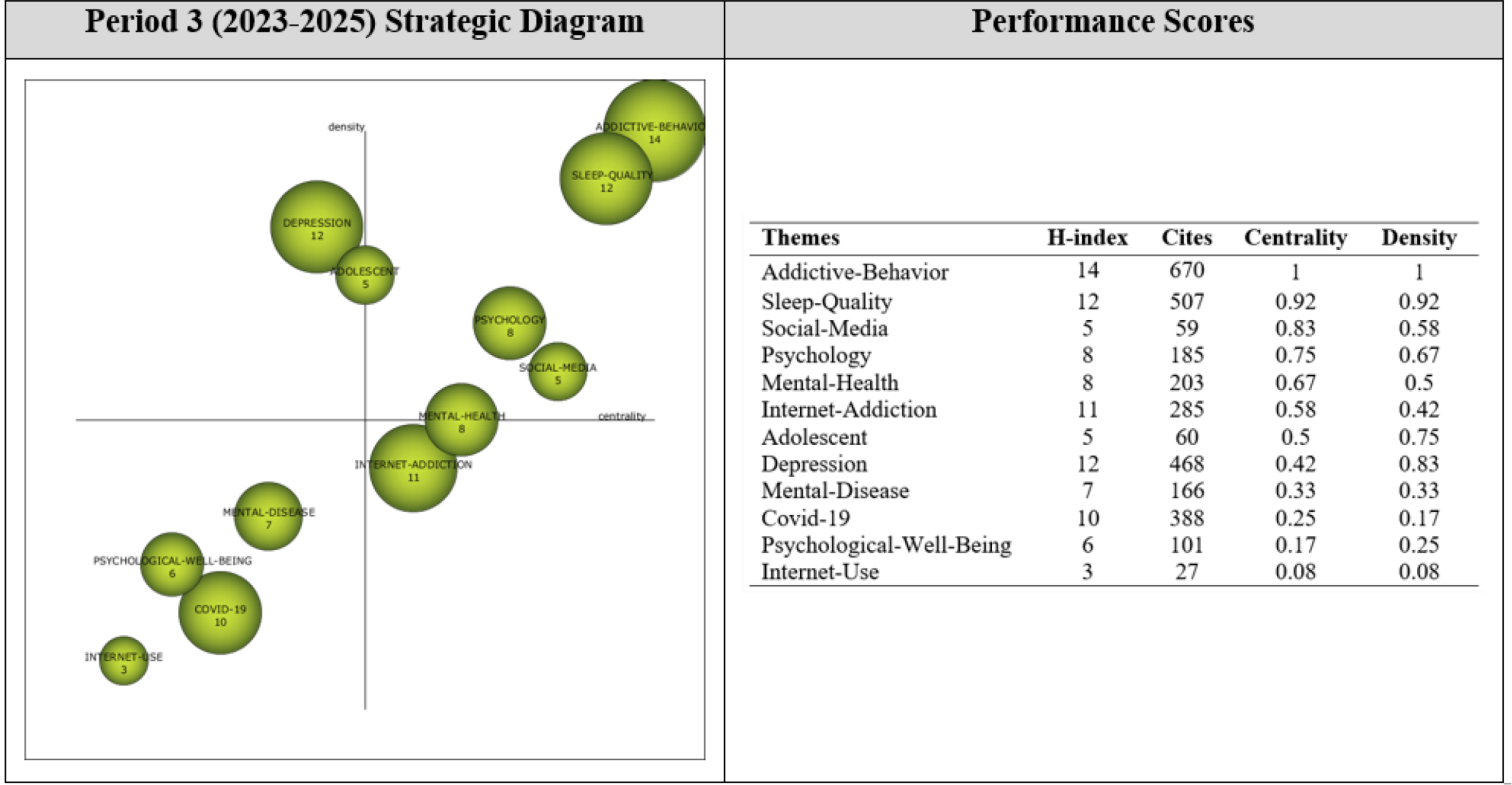
Strategic themes of Period 3 (2023-2025).

Analysis of 430 articles published during Period 3 revealed 12 themes that guided research during this period. As shown in **Figure 9**, the most significant themes (i.e., the motor themes) were “Addictive-Behavior”, “Sleep-Quality”, “Social-Media”, “Psychology”, “Mental-Health”, and “Adolescent”.

During the third period (2023-2025), DDICTIVE-BEHAVIOR, SLEEP-QUALITY, SOCIAL-MEDIA, PSYCHOLOGY, MENTAL-HEALTH, and ADOLESCENT were among the motor themes with their high centrality and density values. These were the themes that contributed to the growth of the mental health-digital addiction research field during this period. The DEPRESSION theme was a highly developed and isolated theme, while the MENTAL-DISEASE, COVID-19, PSYCHOLOGICAL-WELL-BEING, and INTERNET-USE themes were among the emerging/declining themes of the period. The INTERNET-ADDICTION theme had higher centrality and lower density value, and thus was located in the basic and transversal themes quartile of the strategic diagram. The theme with the highest H-index value was the ADDICTIVE-BEHAVIOR theme.

**Figure 10:**
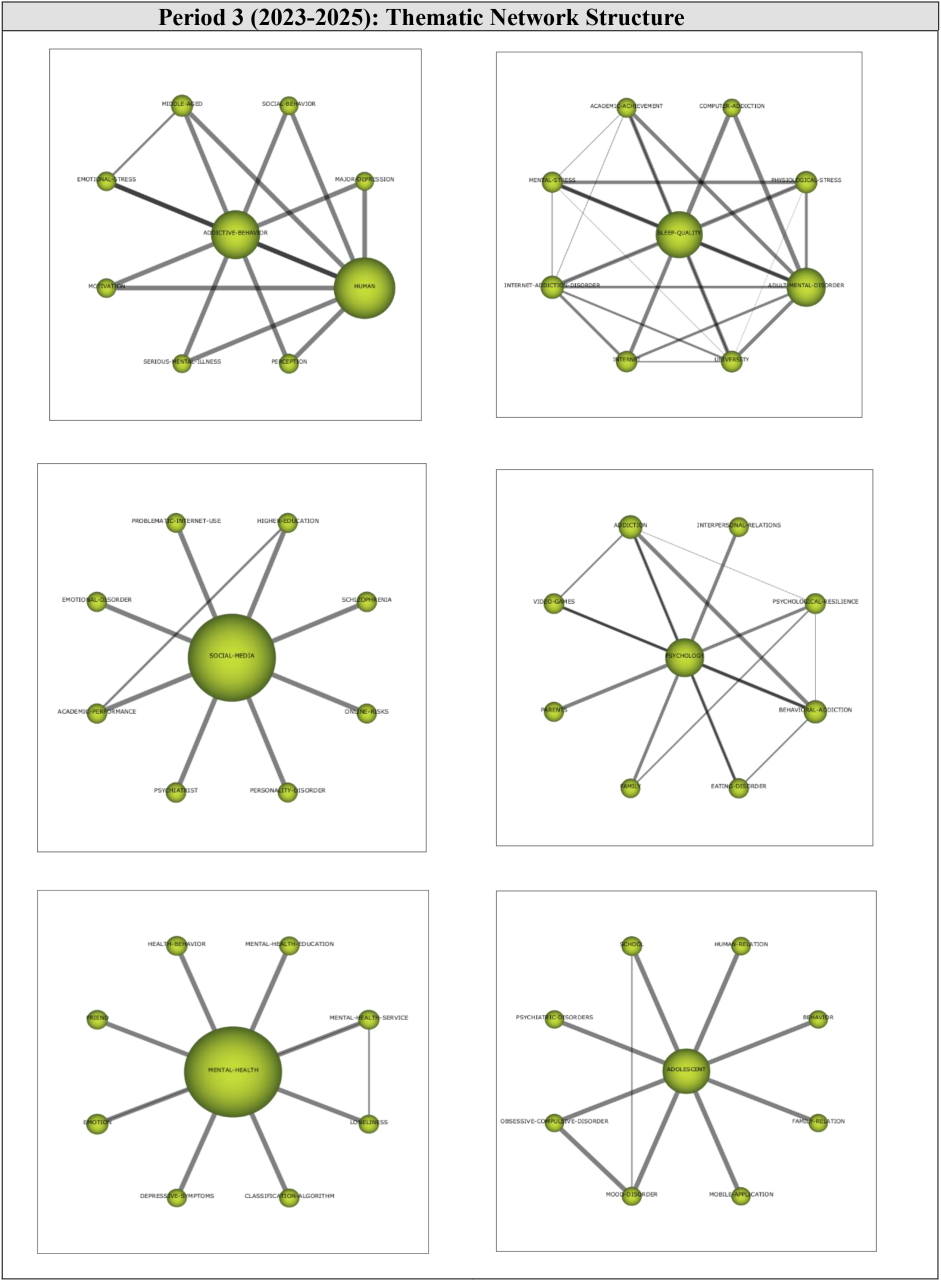
Thematic cluster networks for Period 3 (2023-2025) motor themes.

Thematic network structures were examined to determine the sub-themes relevant to the motor themes of the third period (2023-2025). The thematic network of each engine theme is shown in **Figure 10**.

The motor theme ADDICTIVE-BEHAVIOR (1, 1) was found to be strongly related to the sub-themes of *Social-Behavior, Major-Depression, Human, Perception, Serious-Mental-Illness, Motivation, Emotional-Stress*, and *Middle-Aged*, all of which were found to be interrelated. These results indicate that this research focused on investigating the addictive behaviors of middle-aged people as well as serious mental illnesses such as major depression.

The motor theme SLEEP-QUALITY (0.92, 0.92) was found to be related to the sub-themes of *Computer-Addiction, Physiological-Stress, Adult-Mental-Disorder, University, Internet, Internet-Addiction-Disorder, Mental-Stress*, and *Academic-Achievement.* Some sub-themes were found to be related to each other. These results show that most studies conducted during this period focused on the relationship between sleep disorders, computer addiction, internet addiction, and academic achievement.

The motor theme SOCIAL-MEDIA (0.83, 0.58) was found to be related to the sub-themes of *Higher Education, Schizophrenia, Online Risks, Personality Disorder, Psychiatrist, Academic Performance, Emotional Disorder*, and *Problematic Internet Use.* The sub-themes of Higher Education and Academic Performance were observed to be interrelated. These results indicate that studies have focused on the mental health risks of internet addiction or the problematic use of the internet. The connection between Higher Education and Academic Performance implies that these studies had a particular interest in how mental health problems and the problematic/excessive use of the internet influenced academic achievement.

The motor theme of PSYCHOLOGY (0.75, 0.67) was found to be related to the sub-themes of *Interpersonal Relations, Psychological Resilience, Behavioral Addiction, Eating Disorder, Family, Parents, Video Games*, and *Addiction*, all of which were interrelated. These results indicate that this research focused on the role of the parent in the addiction-mental health relationship, as well as an interest in psychological factors regulating or being influenced by their interaction.

The motor theme of MENTAL HEALTH (0.67, 0.5) was found to be associated with the sub-themes of *Mental Health Education, Mental Health Service, Loneliness, Classification Algorithm, Depressive Symptoms, Emotion, Friend*, and *Health Behavior.* The Mental Health Service and Loneliness sub-themes were observed to be interrelated. These results indicate that research on mental health is focused on social issues such as loneliness and friendship, as well as mental health services and education.

The motor theme, ADOLESCENT (0.5, 0.75), was found to be associated with the sub-themes of *Human Relation, Behavior, Family Relation, Mobile Application, Mood Disorder, Obsessive-Compulsive Disorder, Psychiatric Disorders*, and *School.* The Mood Disorder and the School sub-themes were found to be interrelated. These results indicate that this line of research has a particular interest in psychological disorders in adolescents.

### 3.3 Thematic evolution analysis

Thematic evolution analysis shows how themes were transformed across periods of analysis, and illuminates the thematic growth of the research field from its birth to maturity.

**Figure 11:**
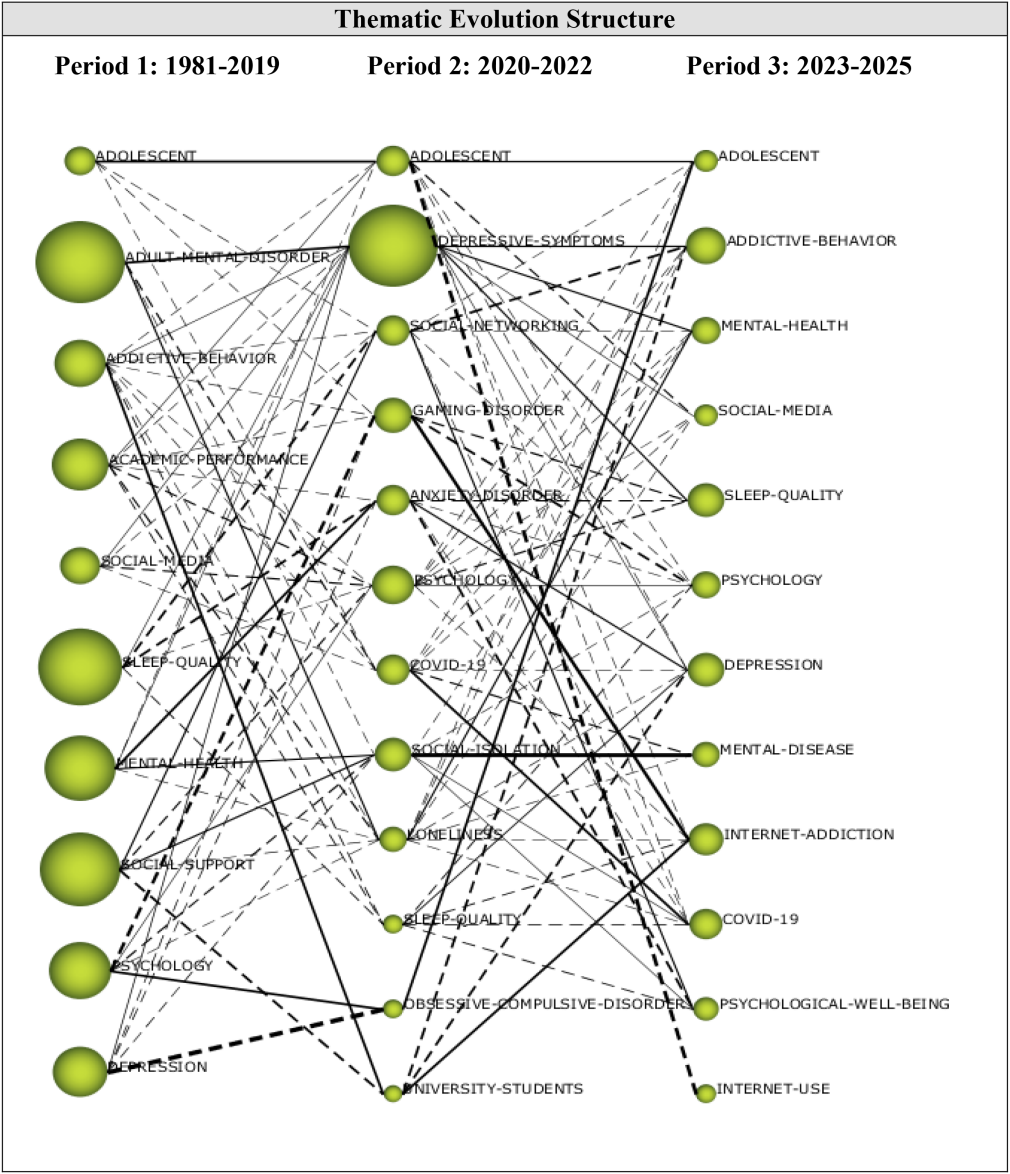
Thematic evolution map.

As shown in the overlapping graph in **Figure 11**, research addressing digital addiction-mental health during Period 1 focused on addictive behaviors and problematic social media use on the digital addiction side, and sleep quality and depression on the mental health side, with particular reference to adolescents, which might also explain their focus on academic performance during Period 2. This focus later shifted to university students and themes like loneliness and social isolation, echoing the influence of the COVID-19 pandemic. From the addiction perspective, the focus was on social networking and gaming disorders, while the focus on mental health was more diversified. Results for Period 3 reflected a mix of themes from both Periods 1 and 2, underscoring the continued research interest in how the problematic use of the internet and social media was linked to addictive behaviors and impaired mental health, with an additional investigation from a psychological well-being perspective.

According to **Figure 10**, the themes of ADOLESCENT, SLEEP-QUALITY, and PSYCHOLOGY were the most enduring themes contributing to the development of the research area. These themes persisted throughout three periods and were also linked to other themes. For example, the ADOLESCENT theme had strong connections with the themes of SOCIAL-NETWORKING, GAMING-DISORDER, and COVID-19 appeared during the second period, while it had a strong link with the themes of SOCIAL-MEDIA, PSYCHOLOGY, DEPRESSION, and INTERNET-USE appeared during the third period. The SLEEP-QUALITY theme was strongly linked to DEPRESSIVE-SYMPTOMS, SOCIAL-NETWORKING, ANXIETY-DISORDER, and PSYCHOLOGY themes, which appeared during the second period, and the PSYCHOLOGY, DEPRESSION, INTERNET-ADDICTION, COVID-19, and PSYCHOLOGICAL-WELL-BEING themes from the third period. The PSYCHOLOGY theme was strongly related to the GAMING-DISORDER, SOCIAL-ISOLATION, LONELINESS, and OBSESSIVE-COMPULSIVE-DISORDER themes, which appeared during the second period, and the ADOLESCENT, ADDICTIVE-BEHAVIOR, MENTAL-HEALTH, SOCIAL-MEDIA, SLEEP-QUALITY, and INTERNET-ADDICTION themes from the third period.

The ADDICTIVE-BEHAVIOR, SOCIAL-MEDIA, MENTAL-HEALTH, and DEPRESSION themes of the first period evolved into different themes during the second period, while reappearing during the third period. The ADDICTIVE-BEHAVIOR theme was found to be strongly related to the ADOLESCENT, DEPRESSIVE-SYMPTOMS, SOCIAL-NETWORKING, GAMING-DISORDER, PSYCHOLOGY, COVID-19, LONELINESS, SLEEP-QUALITY, and UNIVERSITY-STUDENTS themes of the second period. The SOCIAL-MEDIA theme was strongly related to the DEPRESSIVE-SYMPTOMS, SOCIAL-NETWORKING, PSYCHOLOGY, and COVID-19 themes of the third period. The MENTAL-HEALTH theme had strong links with the DEPRESSIVE-SYMPTOMS, ANXIETY-DISORDER, SOCIAL-ISOLATION, and LONELINESS themes of the second period. The DEPRESSION theme of the first period was linked to the DEPRESSIVE-SYMPTOMS, GAMING-DISORDER, ANXIETY-DISORDER, SOCIAL-ISOLATION, and OBSESSIVE-COMPULSIVE-DISORDER themes of the second period.

The ADULT-MENTAL-DISORDER theme of the first period was strongly related to the DEPRESSIVE-SYMPTOMS, COVID-19, SOCIAL-ISOLATION, and LONELINESS themes of the second period. The ACADEMIC-PERFORMANCE theme of the first semester was linked to the ADOLESCENT, DEPRESSIVE-SYMPTOMS, GAMING-DISORDER, ANXIETY-DISORDER, PSYCHOLOGY, LONELINESS, and SLEEP-QUALITY themes of the second period. The SOCIAL-SUPPORT theme of the first period was strongly linked to the SOCIAL-NETWORKING, PSYCHOLOGY, SOCIAL-ISOLATION, LONELINESS, and UNIVERSITY-STUDENTS themes of the second period. The DEPRESSIVE-SYMPTOMS theme from the second period had a strong relationship with the ADDICTIVE-BEHAVIOR, MENTAL-HEALTH, SOCIAL-MEDIA, SLEEP-QUALITY, DEPRESSION, and COVID-19 themes of the third period.

The SOCIAL-NETWORKING theme of the second period was strongly linked to the ADOLESCENT, ADDICTIVE-BEHAVIOR, MENTAL-HEALTH, PSYCHOLOGY, COVID-19, and PSYCHOLOGICAL-WELL-BEING themes of the third period. The GAMING-DISORDER theme was strongly linked to SLEEP-QUALITY, PSYCHOLOGY, and INTERNET-ADDICTION themes of the third period. The ANXIETY-DISORDER theme from the second period was strongly related to the SLEEP-QUALITY, PSYCHOLOGY, DEPRESSION, COVID-19, and PSYCHOLOGICAL-WELL-BEING themes of the third period. The COVID-19 theme from the second semester persisted into the third period, forming strong connections with ADDICTIVE-BEHAVIOR, MENTAL-HEALTH, SOCIAL-MEDIA, DEPRESSION, and MENTAL-DISEASE themes. The SOCIAL-ISOLATION theme from the second semester also had strong connections with MENTAL-HEALTH, PSYCHOLOGY, MENTAL-DISEASE, COVID-19, and PSYCHOLOGICAL-WELL-BEING themes of the third period.

The LONELINESS theme from the second period was strongly linked to the ADOLESCENT, ADDICTIVE-BEHAVIOR, MENTAL-HEALTH, SOCIAL-MEDIA, DEPRESSION, INTERNET-ADDICTION, MENTAL-DISEASE, and COVID-19 themes of the third period. The OBSESSIVE-COMPULSIVE-DISORDER theme of the second period was strongly linked to the ADOLESCENT theme of the third period. The UNIVERSITY-STUDENTS theme of the second period was strongly linked to the ADDICTIVE-BEHAVIOR, DEPRESSION, and INTERNET-ADDICTION themes of the third period.

## 4 Discussion

This study analyzed the conceptual and intellectual development of research addressing the relationship between digital addiction and mental health. By exploring the conceptual and intellectual development of the digital addiction-mental health research field through period-based science-mapping analyses, this study revealed the key research themes underpinning its development. It provided significant results that can guide future investigations and contribute to the development of the theoretical framework regarding the digital addiction-mental health relationship.

Overall, the analysis yielded results that are concentrated on two main axes: themes related to digital addiction and those related to mental health. More specifically, research conducted during the first period of analysis(1981-2019) addressed the theme of mental disorders in adults, focusing on its connections with such relevant themes as psychiatrists, anxiety disorders, loneliness, depressive symptoms, emotions, family relationships, and health behavior. This implies that research published during the initial period focused on the causes and consequences of mental disorders in adults. Current evidence shows that individual, interpersonal, and behavioral processes are effective together in the emergence and maintenance of mental disorders in adults. For example, personality traits (44) and negative family relationships (45) play a significant role in disorders such as depression and anxiety disorders are also a condition that is highly co-occurring with mental disorders in individuals (46). Similarly, loneliness is effective in the emergence and development of mental disorders in adults (47). In addition, various pieces of evidence are presented suggesting that healthy lifestyle behaviors (adequate and balanced nutrition, sufficient and regular sleep, regular and sufficient exercise, etc.) can provide protection against various mental disorders (48). Research investigating relationship between digital addiction and mental health seems to be guided by these existing results in the broader mental health literature across all three periods of analysis.

### 4.1 Period 1 (1981-2019)

The results from the analysis of documents published during the first period (1981-2019) showed that adult mental disorders, mental health, sleep quality, academic performance, and social support were the most influential themes in digital addiction and mental health research. The current literature provides significant evidence that the relationship between digital addiction and mental disorders in adults is complex and interactive (10, 26). Furthermore, the impact of problematic internet, social media, and smartphone use on adults’ sleep quality is increasingly emphasized, and many studies were conducted to explore the relationship between sleep quality, digital addiction, and mental health. In particular, time spent in the digital world until late at night negatively affects sleep duration and quality, which are associated with increased levels of depression, anxiety, and stress (49–51).

Academic performance was another influential theme during the first period. Studies show that the problematic use of digital technologies, particularly among university students, can significantly reduce academic performance. For example, Ning & Inan (52) reported that social media addiction negatively affects the academic performance of university students, while having a positive effect on anxiety and stress. Abbasi et al. (53) reported that smartphone addiction for entertainment and gaming purposes reduces academic performance while use related to classes had a positive effect Furthermore, research shows that digital addiction not only negatively affects individuals’ academic performance but also has far-reaching effects on mental health (depression, anxiety, distress, sleep disorders, etc.) (54, 55), with possible harm to their academic achievement. These results demonstrate that academic performance is multidimensionally linked to addictive behaviors and mental health indicators.

As can be expected, “mental health” was among the prominent themes of the first period. The cluster network of mental health themes showed that it was mostly addressed in relation to social isolation, computer addiction, university students, parents, anxiety disorders, behavior, and smartphone addiction. Research indicates that social isolation is a significant determinant in understanding the relationship between digital addiction and mental health among students. Loneliness and a lack of social connection are considered important factors in problematic digital device use and online platform orientation, with individuals attempting to compensate for the lack of social support in this way (56). This situation increases the tendency for problematic phone and internet use while also causing increases in depression, anxiety, and stress levels (26, 57, 58). These results show that digital addiction can affect mental health both directly and indirectly, and that research on students’ mental health focuses on smartphone and computer addiction. Social support was one of the prominent issues in digital addiction and mental health research during the first period. Research emphasized that social support mechanisms played a crucial role as a buffer in regulating the complex interplay between digital addiction and mental health. For example, individuals with adequate levels of social support were more resilient to the negative effects of problematic internet and social media use, with a lower risk of anxiety and depression (59, 60).

### 4.2 Period 2 (2020-2022)

During the second period of analysis involving documents published between 2020 and 2022, results indicated that the knowledge base on digital addiction and mental health expanded, focusing on themes such as depressive symptoms, social isolation, anxiety, and gaming disorders. During this period, depressive symptoms emerged as the most studied theme. One reason for the prominence of depressive symptoms in digital addiction and mental health research may be that depressive symptoms are an important factor in explaining the relationship between digital addiction and general mental health in adults. The relevant literature indicates that individuals with high levels of depressive symptoms are more prone to problematic internet and social media use to cope with stress and negative emotions (29, 61, 62).

The social isolation theme was among the significant themes that attracted researchers’ interest during the second period. Social isolation is a significant risk factor for the development of digital addiction and the emergence of mental health problems in individuals (60, 63). A possible reason why the theme of social isolation was so prominent could be that socially isolated individuals are more likely to resort to online digital environments, which might also lead to mental health problems by triggering depression and anxiety (58, 64).

Anxiety disorder is another important theme that received increasing research interest during the second period. Anxiety disorder is a psychiatric condition characterized by excessive and persistent anxiety, restlessness, tension, and avoidance responses to situations where there is no real threat (65, 66). Research shows that individuals with high anxiety levels use digital environments more intensively to avoid anxiety, find emotional relief, or distance themselves from social threats (67, 68). This may reduce anxiety in the short term, but in the long term, it increases the tendency towards addiction and negatively affects mental health (69).

Another significant theme that emerged during the second period was gaming disorder. This corresponds with the World Health Organization’s inclusion of gaming disorder in the mental disease diagnoses category in 2019. With the proliferation of smartphones, the accessibility of online games, and the gaming industry creating a huge economy, gaming disorder is attracting increasing attention within digital addiction literature (70, 71). Gaming disorder, defined as a problem characterized by excessive, uncontrollable gaming behavior, is particularly associated with psychological problems such as depression, anxiety, social withdrawal, and decreased life satisfaction in young adults (72, 73).

The results covering the final analysis period, from 2023 to 2025, showed that the theme of addictive behavior was the most influential theme contributing to the growth of the digital addiction and mental health research field. However, while sleep quality and mental health remained important as driving themes, social media, psychology, and adolescence emerged as three other significant new themes driving research. The increasing use of digital technologies for work and socialization/entertainment over the last decade has made digital addiction a more prominent topic in academic discussions. Restrictions during and after the pandemic have permanently increased individuals’ online interactions, screen time, and the frequency with which they use digital tools. This has led to addictive behaviors and, consequently, an increase in mental health problems such as anxiety, depression, and social isolation. Therefore, the theme of “addictive behavior” has attracted significant attention from researchers during this period (74).

### 4.3 Period 3 (2023-2025)

The science mapping analysis revealed that research published during the third period focused on social media, psychology, and adolescence themes. The rapid proliferation of social media applications such as TikTok, Reels, and similar platforms, particularly among adolescents, since the mid-2020s, has drawn researchers’ attention to this area (75, 76). Increasingly problematic social media use among adolescents has begun to transform into addictive behaviors, ultimately leading to depression, anxiety, and other psychological symptoms (77–79).

It is also noteworthy that addictive behavior emerged as an independent theme only during the third period. This may indicate that digital addiction behavior is accepted as a general concept encompassing addictions to different types of digital media. However, further studies are needed to deepen our understanding and further elaborate on the concept. Given the continuous diversification of digital technologies, it is likely that some other types of addiction related to the problematic or excessive use of digital technologies or platforms have yet to be discovered. Peñafiel et al. (80) suggest that addressing all forms of digital addiction as a single general pathology would help develop a more comprehensive framework for prevention, diagnosis, and treatment.

## 5 Conclusion

This research has enriched the literature by clarifying the intellectual foundations and conceptual framework of studies examining the link between digital addiction and mental health. The findings suggest several implications for both theory and practice.

Science-mapping analyses across three consecutive periods of research addressing mental health and digital addiction showed that adult mental disorders, sleep quality, academic performance, mental health, and social support were the most influential themes between 1981 and 2019, while depressive symptoms, social isolation, anxiety, and gaming disorder garnered more research interest between 2020 and 2022. More recently, i.e., between 2023-2025, addictive behavior, sleep quality, social media, psychology, and adolescent themes have guided the development of its knowledge base. However, we should also note the emergence of “addictive behavior” as a theme represents a shift in research trends rather than an established clinical diagnostic consensus in psychiatry.

The current results show that studies on the link between digital addiction and mental health are nourished by existing theoretical foundations and evidence in the literature of both fields. A detailed examination of the themes and sub-themes revealed that research focused on five types of digital addiction: smartphone addiction, social media addiction, video game addiction, internet addiction, and computer addiction. Furthermore, in the current analysis, adult mental disorders, sleep quality, mental health, symptoms of depression, anxiety disorders, gaming disorders, addictive behavior, and psychology emerged as common themes related to mental health. A closer examination of sub-themes showed that physiological stress, mental stress, loneliness, major depression, emotion, social isolation, schizophrenia, mental illness, psychological resilience, psychological well-being, serious mental illness, and emotional stress frequently appeared in mental health research.

## 6 Limitations

Despite its significant contribution, the study bears some limitations that should be considered while interpreting its findings. First, the study differs from classic reviews that aim to synthesize findings to produce general conclusions in that it conducts a bibliometric and conceptual analysis of published research to highlight research trajectories that are sufficiently or insufficiently taken in the field. Therefore, the present analysis aims to describe the intellectual and conceptual development of the field rather than providing synthesized findings on how digital addiction influences mental health. Particularly, interpreting these results as evidence of a ‘clinical consensus’ that addictive behaviors constitute a unified general pathology might be misleading, and would likely require additional empirical clinical support. Similarly, a cautious and nuanced approach should be adopted when interpreting the emergence of “addictive behavior” as a core theme since this mainly indicates trends in scholarly discussion and conceptual development rather than directly referring to an established diagnostic consensus in psychiatry field.

Additionally, the dataset and relevant metadata are limited to those provided by WoS and Scopus and may have missed relevant data available on other research platforms. In addition, the analysis was restricted to English-language publications, which may have led to the exclusion of relevant studies published in other languages. This is particularly important in the context of digital addiction research, where a substantial body of work originates from regions such as East Asia. As a result, the findings may underrepresent region-specific perspectives, theoretical approaches, and empirical patterns developed in non-English-speaking contexts. In addition, the concentration of publications from relatively high-income countries suggests a potential geographical imbalance in the dataset, which might have influenced the intellectual structure identified in the analysis since research priorities, access to technology, and patterns of digital use can vary significantly across socio-economic and cultural contexts. Therefore, the generalizability of the findings should be interpreted with caution, particularly when extending conclusions to underrepresented regions. Future research could address this limitation by incorporating multilingual databases and region-specific sources to provide a more globally inclusive understanding of the field.

## Data Availability

The original contributions presented in the study are included in the article/supplementary material, Further inquiries can be directed to the corresponding author.
